# Germline-Transmitted Genome Editing in *Arabidopsis thaliana* Using TAL-Effector-Nucleases

**DOI:** 10.1371/journal.pone.0121056

**Published:** 2015-03-30

**Authors:** Joachim Forner, Anne Pfeiffer, Tobias Langenecker, Pablo Manavella, Jan U. Lohmann

**Affiliations:** 1 Centre for Organismal Studies, Heidelberg University, Heidelberg, Baden-Württemberg, Germany; 2 Department of Molecular Biology, Max Planck Institute for Developmental Biology, Tübingen, Baden-Württemberg, Germany

## Abstract

Transcription activator–like effector nucleases (TALENs) are custom-made bi-partite endonucleases that have recently been developed and applied for genome engineering in a wide variety of organisms. However, they have been only scarcely used in plants, especially for germline-modification. Here we report the efficient creation of small, germline-transmitted deletions in *Arabidopsis thaliana* via TALENs that were delivered by stably integrated transgenes. Using meristem specific promoters to drive expression of two TALEN arms directed at the *CLV3* coding sequence, we observed very high phenotype frequencies in the T2 generation. In some instances, full *CLV3* loss-of-function was already observed in the T1 generation, suggesting that transgenic delivery of TALENs can cause highly efficient genome modification. In contrast, constitutive TALEN expression in the shoot apical meristem (SAM) did not cause additional phenotypes and genome re-sequencing confirmed little off-target effects, demonstrating exquisite target specificity.

## Introduction

During the last decades, considerable resources for reverse genetics in the reference plant *Arabidopsis thaliana* (*A*. *thaliana*) have been developed, including T-DNA insertion collections like the SALK [1] and SAIL lines [2], cDNA libraries that can be used for RNAi expression (e.g. AGRIKOLA [3]) or the artificial micro RNA [4], MIGS [5] and TILLING [6] techniques. These tools allow studying the function of individual genes or groups of genes by analyzing complete or partial loss-of-function phenotypes and thus are indispensible for modern plant biology. Consequently, a large number of research articles in the field contain data acquired using these resources. However, all these approaches have inherent limitations. For example, suitable mutant alleles for the gene of interest might not be available in the public T-DNA insertion collections due to insertion bias. Since T-DNA mutagenesis relies on the integration of large pieces (5kb) of foreign DNA, which include potent promoter and enhancer sequences to drive selection cassettes, the effects of T-DNA integration events on the function of nearby genes is difficult to predict or analyze. Furthermore, these resources are only available for very few accessions of *A*. *thaliana*, precluding efficient genetic analysis in most ecotypes. Alternative strategies, such as, RNA interference-based techniques commonly fail to deliver a full knock-down and suffer from off-target effects. Thus, there is substantial demand for targeted genome editing techniques to deliver more specific gene modifications in *A*. *thaliana* and related species. One attractive route to this end is the use of site-specific endonucleases that create double-strand breaks at the target site, which in turn will be repaired by the cellular machinery using error-prone non-homologous end-joining (NHEJ). Furthermore, creating double-strand breaks in the genome is known to increase the frequency of gene-targeting via homologous recombination by several orders of magnitude and forms the base for genome engineering in a variety of organisms [7,8] opening up this path also for *A*. *thaliana*, which until now has proven to be quite recalcitrant in this aspect [9]. Thus, the delivery of efficient site-specific endonucleases to target tissues in *A*. *thaliana* forms the basis for a whole range of genome editing strategies and substantial progress has been made in recent years.

The meganuclease I-SceI was the first endonuclease used for eukaryotic genome engineering based on induced double-strand breaks [10], which was also successfully applied to plants [11]. However, since the target site of I-SceI is fixed and not present in the *A*. *thaliana* genome, it can only act on a transgene inserted previously and not an arbitrary endogenous sequence. The same constraint applies for alternative meganucleases, and new specificities for these are extremely difficult to engineer and limited to sequences similar to the original meganuclease cutting site [12] [13].

The first fully engineered endonucleases, which allowed full control over targeted recognition sites, were the zinc finger nucleases (ZFNs). ZFNs are chimeric proteins composed of sequence-specific DNA-binding domains, the zinc fingers (ZF), and the FokI endonuclease domain [14]. C2H2 zinc fingers motifs are frequently found in eukaryotic transcription factors and each ZF binds to a specific DNA triplet. For ZFNs, several ZFs are combined into a single protein to increase specificity. The FokI endonuclease at the C-terminus of the ZFNs is only active as dimer, thus for creating a double-strand break two ZFNs must bind in tail-to-tail orientation to bring two FokI monomers together, which further increases specificity. ZFNs have been successfully employed for genome engineering in a variety of organisms [15] [16] [14], including *A*. *thaliana* [17] [18]. However, since the binding specificity of an individual ZF depends on the protein context into which it is embedded, combining individual ZFs to build a new ZFN with a defined DNA-binding site is a non-trivial task, making the whole design process to engineer new specificities cost-, labor- and time-intensive.

The clustered regularly interspaced short palindromic repeats (CRISPR)/CRISPR-associated (CAS) system derived from a bacterial adaptive immunity mechanism against foreign DNA is the latest approach for targeting endogenous genes with endonucleases [19] [20]. Here only one invariant protein (CAS9) is needed for all targets. The specificity is provided by a short RNA sequence, the so-called single guide RNA (sgRNA), which is very easy to engineer. The sgRNA guides the CAS9 endonuclease to the DNA target via sequence homology of the first ~20 nucleotides and a double-strand break is subsequently introduced into the DNA by the activity of CAS9. The CRISPR/CAS9 system has also been successfully applied to plants [21], mainly transiently in protoplasts or via infiltration with agrobacteria, but in some approaches stable transgenic plants have been recovered [22]. A potential problem when working with CRISPR/CAS9 could arise from its mismatch tolerance at the 5’-end of the guide sequence, thus decreasing the effective length of the target sequence and increasing the risk of off-target effects [21].

TALE nucleases (TALENs) [23] are based on transcription activator-like effectors (TALEs) [24]. TALEs are virulence factors of phytopathogenic *Xanthomonas* strains that induce expression of specific genes in the plant host nucleus. Their specificity for defined target sequences is conveyed by a stretch of nearly identical repeat units of 34 amino acids which differ among only at positions 12 and 13, the so called repeat-variable di-residues (RVD). Each repeat binds to one nucleotide on the DNA, and each RVD is specific for only one of the DNA bases. TALENs are created by replacing the TALE C-terminus containing the transcriptional activation domain with a FokI endonuclease domain monomer and usually deleting the N-terminus, thus creating a site-specific endonuclease. The 1:1 correlation between RVD and DNA base is also kept in artificial repeat assemblies, allowing the engineering of TALENs against virtually any chosen sequence, provided it starts with a thymine. Since FokI only works as a dimer two TALEN arms need to bind in close proximity to cleave one locus, greatly improving specificity, although single chain TALEN variants can also be engineered [25]. The binding sites for the single TALENs of a pair need to be separated by a spacer of approximately 14 nucleotides (depending on the exact TALEN design) and the proteins need to bind in a tail-to-tail orientation so that the two FokI moieties come into contact and can create a double strand break. The most N-terminal repeat unit of the TALEN binds the most 5’-base of the binding site.

Despite their rather recent development, TALENs have been extensively used for genome editing in a variety of organisms [26]. However, there is only a limited number of reported applications in plants, especially when it comes to stably modified transgenic lines [27]. For the *SurA*/*SurB* locus in tobacco, targeted knock-outs and YFP knock-ins have been achieved with TALENs, both on the protoplast and callus level [28] but no stable transgenic plants have been regenerated. In rice, callus-derived plants resistant against bacterial blight have been obtained by deleting a short promoter element via TALENs [29]. Targeting the *ADH1* and *NATA2b* loci in transgenic *A*. *thaliana* using estradiol-inducible TALENs, a mutant allele was detected in 0–12% of the T2 plants [30].

Here we report about using TALENs to create heritable *CLV3* loss-of-function mutations in *A*. *thaliana* with efficiencies of up to 100% mutant offspring in the T2 generation, by far exceeding the rate obtained previously [30]. The TALEN expression cassettes under control of different promoters were stably integrated into the target *A*. *thaliana* genome via agrobacterium-mediated transformation. We noted no obvious side-effects of constitutive TALEN expression in the shoot apical meristem (SAM), and in a fully sequenced mutant plant only little off-target effects on the genome were observed.

## Results

To develop efficient and specific genome editing strategies, we chose the *CLV3* locus as a test case. *CLV3* is a small gene, but member of a large gene family on the one hand severely limiting TALEN design options and on the other hand providing an ideal background to test for editing specificity. Furthermore, the *clv3* loss-of function phenotype is well analyzed and with its broadened stem and distinctly misshaped club-like siliques [31] [32] easy to recognize even when grown among hundreds of wild-type plants. We hypothesized that depending on the efficiency of the TALEN induced double stranded DNA break, plants should either slowly develop the *clv3* mutant phenotype over the course of their life in the T1 generation, or show the phenotype in T2.

### First generation TALENs

**Fig 1:**
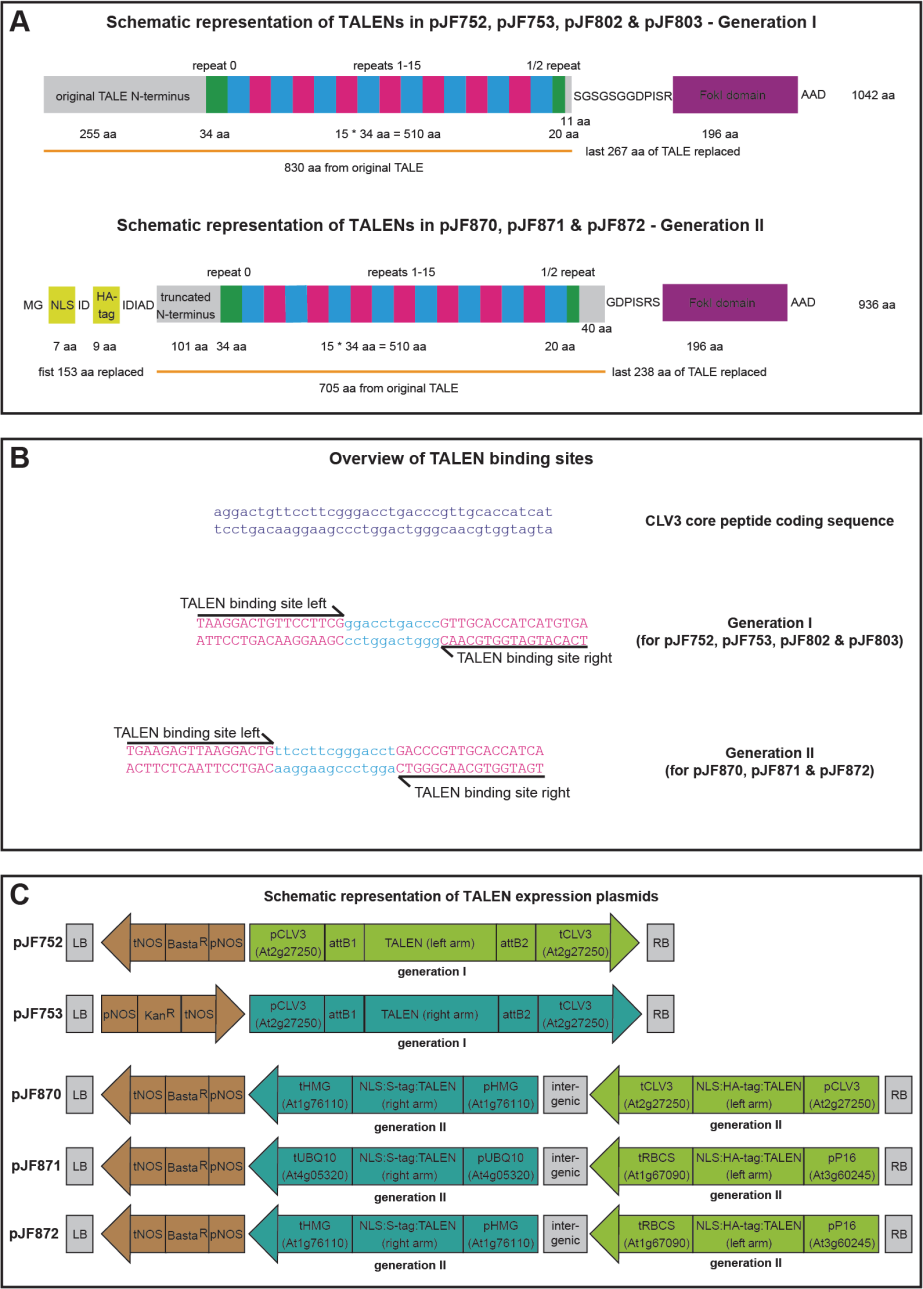
(A) TALEN structure. The variant 34 amino acid (aa) repeats each binding a defined nucleotide as specified by their repeat-variable diresidues are depicted as cyan and magenta boxes. The invariant first and last repeats are marked in green. The part originating from the original TALEs is marked by an orange line. This part and the FokI domain are drawn to scale. For the first generation TALENs, one amino acid (alanine) has been inserted between aa1 and aa2 to create an NcoI site. The S-tag versions are 6 aa longer. (**B**) Binding sites of the TALENs used in this study. (**C**) Constructs for TALEN expression. pJF802 & pJF803 are built similarly to pJF752 & pJF753 but contain the *P16* promoter instead of the *CLV3* promoter and the *RBCS* terminator instead of the *CLV3* terminator. LB: left border. RB: right border. p: promoter. t: terminator. attB1/2: attachment sites from Gateway cloning. Basta^R^: phosphinotricine acetyl transferase. Kan^R^: neomycin phosphotransferase. NOS: nopaline synthase. NLS: nuclear localization signal.

**Fig 2:**
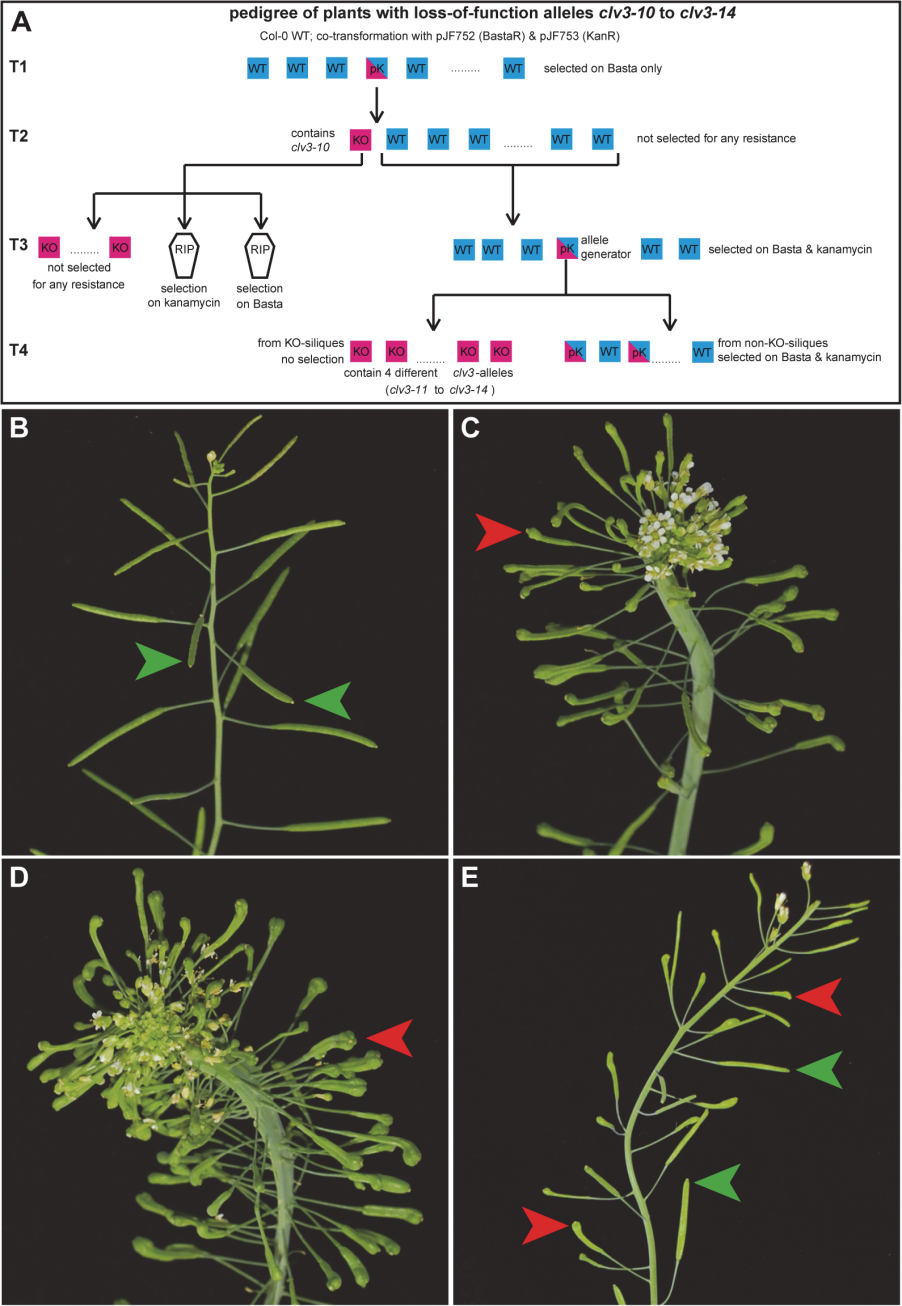
(A) Origin of *clv3-10* to *clv3-14* alleles. Cyan square (WT): plant with no visible sign of *clv3* loss-of-function phenotype. Magenta square (KO): plant with full *clv3* loss-of-function phenotype. Dual-colored squares (pK): plants displaying partial loss-of-function phenotype. RIP: no surviving seedlings. (**B-E**) Inflorescence of Col-0 wildtype (**B**), *clv3-7* (**C**), a *clv3-10* T4 plant displaying the full loss-of-function phenotype (**D**) and a T4 descendant of the pJF752/753 “allele generator” T3 plant displaying a slow onset of the mutant phenotype (**E**). Green arrowheads: wild-type silique; red arrowheads *clv3* mutant silique.

**Fig 3:**
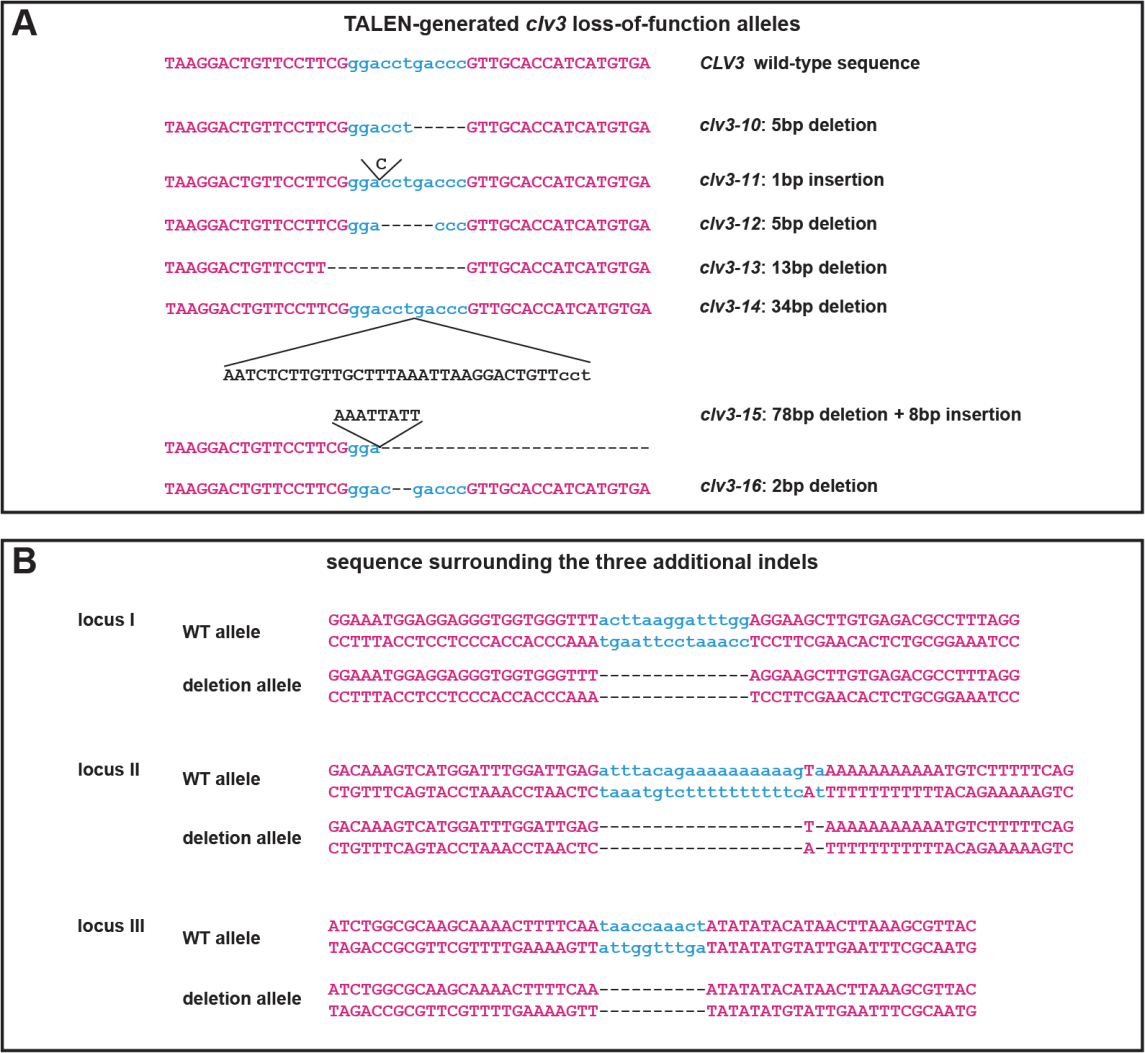
(A) Nucleotide sequences of *clv3-10* to *clv3-16*. TALEN binding sites in magenta, spacer area in cyan. (**B**) Nucleotide sequences surrounding the three additional deletions found in the T2 plant homozygous for *clv3-10*. Cyan lowercase letters in the wild-type sequences represent the nucleotides lost in the deletion alleles.

Our initial experiments were carried out using first generation TALENs commercially produced by Cellectis (Fig. 1A). The two TALEN arms were designed to bind to sequences flanking the sequence encoding the CLV3 core peptide [33] (Fig. 1B) so that essentially any DNA change in this region should result in a loss-of-function phenotype. Each TALEN arm was encoded on a separate T-DNA and both transgenes were driven by the stem cell-specific *CLV3* promoter (Fig. 1C). *A*. *thaliana* Col-0 wild-type plants were co-transformed with both constructs and resulting T1 seeds were selected for only one of the T-DNAs conferring resistance to the herbicide Basta (phosphinotricine) (Fig. 2A). Among approximately 120 T1 plants of which up to 30% are expected to also contain the second T-DNA [34] no plant showed the full *clv3* mutant phenotype. One plant however had a few siliques displaying *clv3* club shaped and curved phenotype. Hundreds of T2 seeds derived from selfing of this plant were sown out without selection and a single T2 plant displayed the full *clv3* phenotype. Genotyping of this plant revealed that it was homozygous for a novel *clv3* allele with a 5 bp deletion between the two TALEN binding sites in the region coding for the CLV3 core peptide, which we called *clv3-10* (Fig. 3A). We concluded that the TALEN dependent double strand break and resulting erroneous repair must have happened in a precursor cell of the pollen and egg cell of the parental T1 plant, but not in an early L2 stem cell [35], since the vast majority of the T2 plants was unaffected.

Having identified a germline transmitted genome-editing event at the desired locus despite the restrictions in TALEN design due to highly related sequences within the gene family, we next aimed to asses the sequence specificity of our approach. To this end genomic DNA from the T2 plant displaying the *clv3* mutant phenotype, as well as from a pool of wild-type plants grown at the same time was prepared and analyzed by next generation sequencing. For the *clv3-10* mutant, we recovered 152 million reads of 50 bp and were able to map 134 million reads to the *A*. *thaliana* reference genome using bowtie2. In addition to the 5bp deletion at the *CLV3* locus we only identified three further deletions in the genome of the *clv3-10* plant, which were not present in our wild-type sample (Fig. 3B). These deletions ranged in size from 10bp to 20bp and were subsequently confirmed by PCR. Interestingly, the sequences surrounding these sites showed no similarity to the TALEN binding sites, suggesting that they might not represent TALEN off-targets, but rather spontaneous mutations. However, only 0.24 deletions are expected to accumulate spontaneously in the four generations passed since the last common ancestor of the T2 *clv3-10* plant and the control wild-type sequenced, assuming a frequency for deletions larger than 3bp of 0.5 x 10^–9^ per site and generation [36]. Thus, we cannot rule out that the observed small deletions in *clv3-10* represent TALEN mediated mutations. Whole genome sequencing of *clv3-10* also did not recover fragments related to our TALEN expressing transgenes, suggesting that these had been segregated away from the *clv3-10* allele.

Next, we analyzed the genetic markup of T3 descendants of the *clv3-10* plant and found that all 20 seedlings showed the *clv3* phenotype, demonstrating that *clv3-10* is a stable and heritable allele. The T3 plants were also all sensitive to kanamycin, the selection marker encoded on the T-DNA for the second TALEN arm, and Basta, confirming the absence of both TALEN transgenes already detected by sequencing.

Having identified a functional TALEN expressing parent, additional T2 siblings of the first knock-out plant were sown out without selection to record phenotypic frequency. However, in the second batch not a single plant showed a full *clv3* mutant phenotype. Therefore, a pool of T3 seeds harvested from these T2 plants was sown out on double-selective plates and six plants resistant to both kanamycin and Basta carrying both TALEN arms were recovered. Five of these never displayed any sign of a *clv3* loss-of-function, whereas in the sixth plant several *clv3* mutant siliques appeared. In addition, the meristem of the primary inflorescence expanded and eventually split, indicating that at least in some sectors a di-allelic mutation of *CLV3* had occurred during postembryonic development. T4 seeds from three club-shaped siliques from the primary inflorescence were sown out and 24 plants randomly selected and propagated. All showed the full *clv3* loss-of-function phenotype. To analyze the genetic make-up of the plants, DNA was isolated from a bulk of their siblings and the *clv3*-locus sub-cloned after PCR-amplification. Sequencing of twelve clones revealed the presence of four new alleles (Figs. 2A and 3A), whereas no wild-type alleles were recovered. This finding implied that the T3 plant had inherited two wild-type *CLV3* alleles from its parent and that the TALENs acted on both alleles during growth of T3 generation. When other T4 seeds derived from parts of the plant which displayed wild-type characteristics were sown out, again on double selective medium to ensure the presence of both TALENs, most T4 plants were wild-type-like in appearance, but some plants repeated the phenotype of the parental T3 plant, i.e. they started to develop club-shaped siliques and broader stems in later stages of development (Fig. 2A and 2E). In general, this phenotype was very variable, ranging from nearly wild-type to almost full *clv3* mutant. The least affected plants developed a single club-like silique at the end of their life, while intermediate plants variably formed club-shaped siliques on individual shoots or even in an asymmetric fashion along a single shoot, consistent with the idea of stem cell derived clonal sectors. The most affected plants displayed the full *clv3* mutant phenotype including fasciation of the stem and club-shaped siliques starting from early stages of inflorescence development. While phenotypic severity varied greatly among individual plants, *clv3*-like phenotypes generally became more prevalent over the course of post-embryonic development, suggesting continued TALEN activity.

Similar results were obtained when the first generation *CLV3* TALENs were driven by the ribosomal protein P16 promoter [37]. 17 T1 plants were selected on Basta only and did not show any phenotype, hundreds of T2 descendants were sown out for each of these plants and only one single plant exhibited a full *clv3* loss-of-function phenotype. Genotyping revealed that this plant carried two different *clv3*-knock-out alleles, namely a 70 bp deletion (*clv3-15*) and a 2 bp deletion (*clv3-16*) (Fig. 3A). T3 descendants were genotyped and plants homozygous for the 70bp-deletion-allele (*clv3-15*) crossed to wild-type. The F1 plants were resistant to kanamycin and Basta, implicating the presence of both TALENs. None of the 24 F1 plants showed any sign of a developing *clv3* phenotype, although each cell had only one intact *CLV3* allele left, which was proven by the recovery of *clv3*-KO F2 plants. Thus, the activity of this TALEN pair under control of the *P16* promoter was very low.

### Second generation TALENs

While we had shown that TALEN mediated editing of the *CLV3* locus is feasible, the frequencies of gene targeting events were generally low. Therefore, we sought to improve TALEN targeting efficiencies to allow the generation of mutants that are more difficult to identify than *clv3*. To this end we employed new TALEN arms derived from second-generation design (Cellectis) and delivered them on a single T-DNA. Using our GreenGate system [38] we constructed a plasmid from which two different promoters drove the two TALEN arms, resulting in transgenic plants which can conveniently be selected with Basta and should have a reduced risk of transgene silencing due to reduction in insertion number and the use of non-overlapping regulatory sequences. To analyze the influence of promoters on targeting efficiencies, we tested three combinations, a meristem-specific one using the *CLV3* (*At2g27250*) and the *At1g76110* promoters [39] on plasmid pJF870, a ubiquitous one with p*P16* (*At3g60245*) [37] and p*UBQ10* (*At4g05320*) in pJF871 and a third version combining the stronger promoters each of the former two combinations with p*P16* and p*At1g76110* in pJF872 (Fig. 1C). After selection of transgenic T1 plants dozens of seedlings per combination were transplanted and screened for signs of the *clv3* loss-of-function phenotype (Table 1, Fig. 2). The large number of individual T1 plants analyzed per line should normalize for potential effects of transgene copy number and therefore expression strength variations on TALEN activity. In case of pJF870, which carried the TALEN arms under the control of two meristem specific promoters, 57 out of the 73 plants developed typical *clv3* defects during development as observed for the T3 and T4 plants of the first generation approach. These plants changed from wild-type morphology to *clv3* mutant phenotype, varying in severity and time of onset. 10 out of the 73 plants exhibited the full *clv3* loss-of-function phenotype already approximately two weeks after germination, judging from the number and density of rosette leaves. During the development of the inflorescence, these plants showed expanded SAMs and produced exclusively club-like siliques. The other six plants were completely wild-type in appearance and did not show any signs of the *clv3* mutant phenotype. For pJF872, which drove expression of the TALEN arms from a very strong ubiquitous and a meristem specific promoter, respectively, the early full *clv3* phenotype was identified in the majority of plants (21 out of 37). In 14 plants, the *clv3* phenotype developed gradually over the life-span and only 2 plants showed no visible signs of a *clv3* mutation at all. However in case of pJF871, which contained two ubiquitous promoters, in most of the T1 plants no *clv3* related morphological changes could be observed (32 out of 44). Nine plants started to develop the *clv3* phenotype, albeit very late in life when compared to pJF870 and pJF872. In further three plants a single or a few club-like siliques were formed. These results showed that by using optimized, second generation TALENs in combination with promoters driving strong expression throughout the SAM, very high gene targeting efficiencies can be achieved leading to di-allelic genome modifications in the first generation.

**Table 1:** T1 phenotypes of pJF870, pJF871 and pJF872 plants.

construct	# of plants with full mutant phenotype from early stage on	# of plants with mutant phenotype gradually building up	# of plants with sporadic club-like siliques	# of plants wild-type like with no signs of *clv3*-loss-of-function	total # of plants examined
pJF870	10 (14%)	57 (78%)	0 (0%)	6 (8%)	73
pJF871	0 (0%)	9 (20%)	3 (7%)	32 (73%)	44
pJF872	21 (57%)	14 (38%)	0 (0%)	2 (5%)	37

The somatic mutagenesis rate was examined for three individual T1 plants from pJF872, which exhibited either wild-type, mild, or strong *clv3* phenotypes (S1 Fig.). DNA was isolated from inflorescence material, the *CLV3* locus around the TALEN target sites amplified and the PCR products subcloned. Plant #15, which displayed no signs of the *clv3* mutant phenotype yielded exclusively wild-type sequences from 16 clones. In contrast, only mutated sequences were recovered from 15 and 18 clones, coming from plants #1 and #3, respectively. Plant #3 showed the full *clv3* knock-out phenotype from early stages on, while plant #1 gradually developed the *clv3* mutant phenotype over time and still had a few wild-type-looking siliques. Therefore, germline transmissible genome modifications seem to go hand in hand with somatic mutations, which can be distinct at the sequence level. Thus, it seems advisable to remove active TALEN transgenes by segregation in subsequent generations to ensure allele stability.

To assess the frequency of germline transmission events, T2 seeds were sown out from ten individual T1 plants and scored for the *clv3* loss-of-function phenotype (Table 2). Four plants had exclusively mutant offspring, indicating 100% efficiency to create offspring with two defective alleles. Three more plants had over 80% mutant descendants and the remaining three plants still produced 10% homozygous *clv3* progeny. These rates indicate that it should be easily possible to identify mutant plants by PCR also for genes not causing a visible loss-of-function phenotype.

**Table 2:** Rate of *clv3*-KO phenotypes among T2 progeny of individual pJF870, pJF871 and pJF872 T1 plants.

construct	plant	phenotype in T1	ratio of ***clv3*** mutant offspring in T2
pJF870	#1	KO phenotype gradually building up	approx. 120 KO (92%): 10 WT (8%): 1 becoming KO
pJF870	#2	KO phenotype gradually building up	approx. 10% of plants KO, rest becoming KO
pJF870	#3	KO phenotype gradually building up	100% KO
pJF871	#1	KO phenotype very slowly building up	approx. 10% KO, rest wild-type like
pJF871	#9	wildtype-like	approx. 10% KO, rest wild-type like
pJF872	#5	KO phenotype gradually building up	82 KO (86%): 13 WT
pJF872	#6	KO phenotype gradually building up	66 KO (86%): 11 WT
pJF872	#16	KO phenotype gradually building up	100% KO
pJF872	#17	full KO phenotype early on	100% KO
pJF872	#18	full KO phenotype early on	100% KO

KO: knock-out; WT: wildtype

## Discussion

Our results demonstrate that transgenic delivery of TALENs allows efficient genomic editing at defined loci with minimal off-target effects. While we only have data on a single locus, the fact that we were able to specifically target a small open reading frame, which is a member of a gene family, suggests that TALENs could generally be suited for unbiased genome modifications.

Nevertheless, we observed substantial variations in targeting efficiency depending on the design of the TALEN arms and the promoters used to drive their expression in planta. Using second generation TALENs in conjunction with promoters that are active throughout the shoot meristem, we were able to generate a large number of independent targeting events for *CLV3*, with mainly small insertions/deletions as expected from error-prone NHEJ repair. Many of these occurred already in the T1, suggesting that bi-allelic double strand breaks had occurred in shoot stem cells at very early stages of development. However, even with a low-efficiency TALEN-promoter combination, we were able to identify in T2 plants with *clv3* loss of function phenotypes, as well as an allele generator plant (Fig. 2A), i.e. a plant producing offspring that reliably create new independent *CLV3* targeting events, with reasonable effort. Furthermore, the identification of an allele generator plant in T3 derived from a phenotypically wild-type parent could be read as an encouraging sign for seemingly failing targeting experiments, since it suggests that screening in T3 or even later might still be effective to isolate the desired genotypes. The high rate of transmission of the mutant alleles to the T2 generation observed in our experiments as compared to Christian et al. is probably due to the choice of promoters [30] (S1 Table). The *At1g76110* promoter is highly active in stem cells [39], as are the *P16* and *CLV3* promoters. Thus, in these plants, the reproductive organs are built from cells that have already been mutated, if efficient TALEN designs are used The 35S promoter, however, is not generally active at high levels, especially not in meristems and young floral organs [40], and possibly also not in the L2 stem cells in the meristem, from which the gametes are ultimately derived. The same holds true for the G10-90 promoter [41]. Also, our target locus, *CLV3*, is actively transcribed only in stem cells and its DNA should therefore be easily accessible for TALENs expressed in these cells. For loci repressed in the meristem or in the reproductive organs and therefore being embedded in more densely packed chromatin, one might expect the efficiencies of creating germline-transmitted mutant alleles to be somewhat lower, although this does not seem to be a problem at least with CRISPR/CAS9 in Drosophila [42].

The activity of the *At1g76110* promoter might also partly explain the substantially higher success rate with the second generation TALENs. However, the new design of the TALEN scaffold, especially the truncation of the N-terminus, might mainly have contributed to this [30].

Taken together, our results suggest that transgenic delivery of TALENs is in principle suited for generating mutants with an efficiency that requires no phenotype-based pre-screening. This would be especially useful for genes for which a desired mutant is not yet known or for mutant combinations where the candidates are closely linked on the genome precluding the combination of existing alleles by crossing. The observation that bi-allelic targeting can occur with high efficiency opens additional important avenues for genetic analyses: TALENs driven from tissue or cell type specific promoters could be used to elucidate gene function in particular cells, or inducible promoters could be used to eliminate genes only at particular developmental stages. Furthermore, it should be very helpful for establishing efficient gene-targeting procedures, such as knock in by homologous recombination, that crucially rely on induced site-specific double-strand breaks [43].

A possible drawback for the routine application of TALENs may be their relatively high price when obtained from a commercial supplier and the technical challenges when assembling them in the lab due to their highly repetitive nature, respectively. However, several mostly Golden Gate-based methods for TALEN assembly have been developed recently, which are fairly cheap and user-friendly, as for example [44] [45], which should alleviate this problem and help to make TALENs a versatile routine tool in plant molecular biology.

## Materials and Methods

### Origin of TALENs

The two TALEN pairs used in this study were purchased from Cellectis bioresearch SAS, Paris, France.

### Plant material, growth, transformation and selection

All experiments were carried out using *Arabidopsis thaliana* accession Col-0. Transgenic plants were generated via a modified floral dip [46] protocol, for which only the tips of the inflorescences are dipped into the agrobacteria solution. Plants were grown under a 16 h/8 h day/night regimen at 22°C and approximately 200 μE light.

For selection of transformants, Basta (glufosinate-ammonium; Bayer CropScience Deutschland GmbH, Langenfeld, Germany) was used at a concentration of 20 mg/L on soil and 10 mg/L on ½ MS plates, respectively. Kanamycin on ½ MS plates was used at 50 μg/mL.

### Whole genome sequencing

DNA was isolated from the tips of the primary and secondary inflorescences of the pJF752/753 T2 knock-out plant containing *clv3-10*, yielding 7.9 ng/μl in 200 μl of which 190 μl were used for sequencing, and a pool of several seedlings from our wild-type, yielding 20.6 ng/μl in 200 μl of which 150 μl were used for sequencing. DNA isolation was done with the DNeasy Plant Mini Kit (QIAGEN GmbH, Hilden, Germany), libraries were prepared using the Illumina TruSeq RNA Sample Preparation Kit v2 according to the manufacturer’s protocol and sequenced on a Illumina HiSeq2000. Sequences were mapped to the TAIR10 genome using bowtie2 (version 2.0.5) [47] with the fast-local option. A total of 56 million and 134 million reads were mapped to the genome for the WT and the TALEN sample, respectively. SNP and INDEL calling was accomplished by using samtools mpileup (version 0.1.16) in combination with bcftools view [48]. Using the variant filter perl script provided by samtools, only variants within regions showing coverage lower than twice the average read depth were considered for further analysis. Variable positions were filtered to have a Phred quality score >30.

### Analysis of mutations in the *CLV3* locus and at possible off-target sites

A part of the *CLV3* locus spanning the TALEN target site was amplified with primers A00954 (5’-tctcgcccttgtaggcttacg-3’) and A00955 (5’-tgcgtatcttacattcacttcagc-3') with an amplicon size of 860 bp in wild-type, A02288 (5’-caaagacgaagggtttaggac-3’) and A02289 (5’-gctgaaagttgtttcttggctg-3’) amplifying 111 bp in wild-type or A00083 (5’-caaggactttccaaccgca-3’) and A00955 yielding 542 bp in wild-type.

The indels at the putative off-target sites of the *clv3-10* T2 plant were amplified with primer pairs A03377 (5’-gactgtgatggtgagaagg-3’) and A03378 (5’-tctccacagctatggcatc-3’) giving an amplicon of 137 bp in wild-type, A03379 (5’-ctctcttctcattggttgtaag-3’) and A03380 (5’-tatttttcgcgtccgctcta-3’) with an amplicon size of 171 bp in wild-type and A03381 (5’- gaaacgattttgaaaattgatgtagg-3’) and A03382 (5’-ttcgatggtacgaattggtcct-3’), encompassing 174 bp in wild-type.

PCR reactions for genotyping were either carried out with GoTaq DNA Polymerase (Promega GmbH, Mannheim, Germany) or using home-made Taq DNA polymerase [49]. The PCR-amplified genomic fragments were either sequenced directly or first subcloned via their A-overhangs into XcmI-digested empty GreenGate entry vectors like pGGA000 [38].

### Plasmid construction

To create pJF752 and pJF753, the TALEN coding sequences were excised from the pTAL.pLess.009633 (left TALEN arm) and pTAL.pLess.009634 (right TALEN arm) plasmids delivered by Cellectis via KpnI and XhoI and ligated into pENTR1A, resulting in pJF634 and pJF635. The final constructs were obtained by LR reactions of these plasmids with pFK317 and pFK321, two pGreen-IIS [50] [51] based binary vectors with *CLV3* promoter and terminator sequences and Basta and kanamycin resistance cassettes, respectively.

pJF870, pJF871 and pJF872 were assembled via GreenGate cloning [38]. NcoI and XbaI sites were inserted into pGGI000 ligating an oligoduplex into the Eco31I-opened plasmid, creating pJF859. In parallel, the internal Eco31I site was removed from pTAL_pLESS_23772 (carrying the right TALEN arm) by replacing the S-tag sequence between the ClaI and Eco32I sites with a modified oligoduplex, yielding pJF858. GreenGate entry modules pGGI009 (left TALEN arm) and pGGI010 (right TALEN arm) were created by excising the TALEN coding sequences from pTAL_pLESS_23770 and pJF858 via NcoI and XbaI and ligation into pJF859. Next, GreenGate supermodules were created. pGGM005 containing p*CLV3*:*HA-tag-TALEN (left)*:t*CLV3*; pGGM006 with p*P16*:*HA-tag-TALEN (left)*:t*rbcS*, pGGN004 providing p*At1g76110*:*S-tag*:*TALEN (right)*:t*At1g76110*; p*NOS*:*Basta* ^*R*^:t*NOS* and pGGN005 with p*UBQ10*:*S-tag*:*TALEN (right)*:t*UBQ10*; p*NOS*:*Basta* ^*R*^:t*NOS*. The M- and N-supermodules where then combined to create pJF870 (pGGM005+pGGN004), pJF871 (pGGM006+pGGN005) and pJF872 (pGGM006+pGGN004).

### Plasmid sequences

Plasmid sequences of the TALEN expression constructs have been deposited at GenBank (http://www.ncbi.nlm.nih.gov/nuccore/) under accession numbers KP293934 to KP293940.

### Availability of newly generated *clv3* loss-of-function lines

Seeds homozygous for the *clv3* mutant alleles of lines *clv3-10* and *clv3-15* and free of the TALEN encoding transgenes have been deposited at the Arabidopsis Biological Resource Center (https://abrc.osu.edu/order-stocks) under stock numbers CS68823 (*clv3-10*) and CS68824 (*clv3-15*), respectively.

## Supporting Information

S1 Fig Determination of somatic mutagenesis rate in pJF872 T1 plants. (TIF)

S2 Fig Flow chart for generating null mutant plants using TALENs. (TIF)

S1 Table Previous reports on heritable mutagenesis in *A*. *thaliana* using engineered endonucleases. (PDF)
