# Correction: Germline-Transmitted Genome Editing in *Arabidopsis thaliana* Using TAL-Effector-Nucleases

**DOI:** 10.1371/journal.pone.0133945

**Published:** 2015-07-20

**Authors:** Joachim Forner, Anne Pfeiffer, Tobias Langenecker, Pablo A. Manavella, Jan U. Lohmann

The fourth author’s name is spelled incorrectly. The correct name is: Pablo A. Manavella. The correct citation is: Forner J, Pfeiffer A, Langenecker T, Manavella PA, Lohmann JU (2015) Germline-Transmitted Genome Editing in *Arabidopsis thaliana* Using TAL-Effector-Nucleases. PLoS ONE 10(3): e0121056. doi:10.1371/journal.pone.0121056
