# Supplementary figures and images for: Germline-Transmitted Genome Editing in Arabidopsis thaliana Using TAL-Effector-Nucleases
Source: PLoS One. 2015 Mar 30;10(3):e0121056. doi: 10.1371/journal.pone.0121056 (PMC4378910; doi:10.1371/journal.pone.0121056)

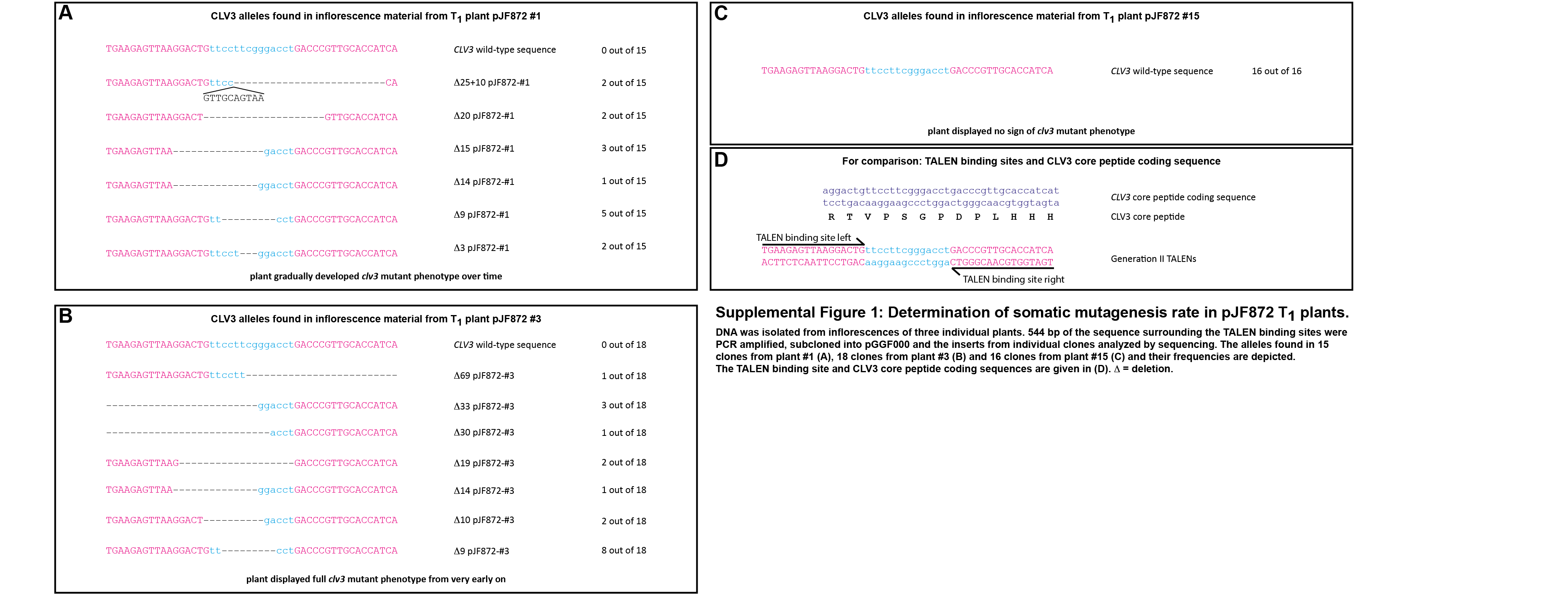

Supplement: S1 Fig — (TIF) [file pone.0121056.s001.tif]

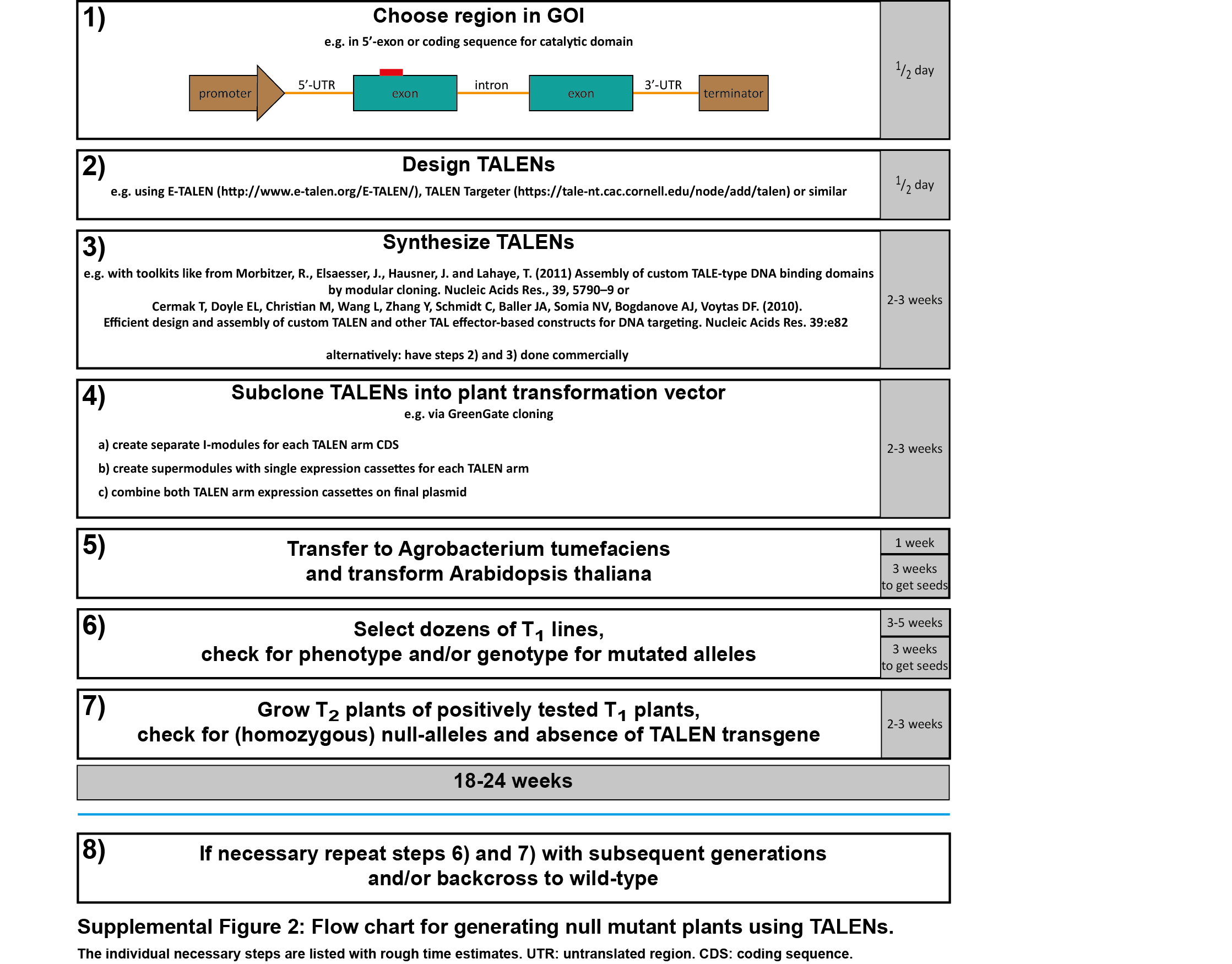

Supplement: S2 Fig — (TIF) [file pone.0121056.s002.tif]
