# Supplementary material for: Germline-Transmitted Genome Editing in Arabidopsis thaliana Using TAL-Effector-Nucleases
Source: PLoS One. 2015 Mar 30;10(3):e0121056. doi: 10.1371/journal.pone.0121056 (PMC4378910; doi:10.1371/journal.pone.0121056)
Supplement: S1 Table — (PDF) [file pone.0121056.s003.pdf]

Supplemental Table 1:

Previous reports on heritable mutagenesis in *A. thaliana* using engineered endonucleases

Study	TALEN/ZFN framework	Promoter used	Target gene	Somatic mutagenesis rate	Germline transmission rate
Christian M, Qi Y, Zhang Y, Voytas DF (2013) Targeted mutagenesis of Arabidopsis thaliana using engineered TAL effector nucleases. G3 (Bethesda) 3: 1697–1705.	N152/C63 TALEN; TALEN arms separated by T2A ribosomal skipping motif	XVE; CaMV 35S	*ADH1*, *TT4*, *MAPKKK1*, *DSK2Ba*, *DSK2Bb*, *NATA2a*, *NATA2b*	2-73%	≤12.0% (*ADH1*; XVE); ≤4.3% (*NATA2b*; 35S) of T_2 progeny harbored mutation
Osakabe K, Osakabe Y, Toki S (2010) Site-directed mutagenesis in Arabidopsis using custom-designed zinc finger nucleases. Proceedings of the National Academy of Sciences 107: 12034-12039.	ZFN, arms separated by T2A ribosomal skipping motif	HSP18.2	*ABI4*	≤2.86%	≤7.3% of T_2 progeny harbored mutation
Zhang F, Maeder ML, Unger-Wallace E, Hoshaw JP, Reyon D, Christian M, Li X, Pierick CJ, Dobbs D, Peterson T, Joung JK, Voytas DF (2010) High frequency targeted mutagenesis in Arabidopsis thaliana using zinc finger nucleases. Proceedings of the National Academy of Sciences 107: 12028-12033.	ZFN, arms separated by T2A ribosomal skipping motif	XVE	*ADH1*, *TT4*	16% (*ADH1*), 7% (*TT4*)	69% (*ADH1*), 33% (*TT4*) of T_1 plants produced mutant offspring
Lloyd A, Plaisier CL, Carroll D, Drews GN (2005) Targeted mutagenesis using zinc-finger nucleases in Arabidopsis. Proceedings of the National Academy of Sciences 102: 2232-2237.	ZFN (QQR)	HSP18.2	QQR binding sites on transgene	1.7–19.6%	9.7% of induced plants produced offspring that harbored mutation

In rice for comparison:

Study	TALEN/ZFN framework	Promoter used	Target gene	Somatic mutagenesis rate	Germline transmission rate
Li T, Liu B, Spalding MH, Weeks DP, Yang B (2012) High-efficiency TALEN-based gene editing produces disease-resistant rice. Nat Biotechnol 30: 390-392.	TALEN with full N-terminus	CaMV 35S & UBI1 (maize)	*Os11N3* (*OsSWEET14*)	n.d.	48-63% of T_1 progeny harbored mutation

ZFN: zinc finger nuclease
